# Legumes in bread baking: A hidden risk for an IgE-mediated inhalant allergy 

**DOI:** 10.5414/ALX02581E

**Published:** 2025-07-15

**Authors:** Sabine Kespohl, Christian Eisenhawer, Silke Maryska, Ingrid Sander, Irene Mittermann, Martina Aumayr, Monika Raulf

**Affiliations:** 1 Institute for Prevention and Occupational Medicine of the German Social Accident Insurance, Institute of the Ruhr University Bochum (IPA), Bochum, Germany, and; 2 MacroAssay Diagnostics, Vienna, Austria

**Keywords:** baker’s asthma, occupational medicine, skin test, specific inhalation challenge, in-vitro IgE diagnosis, identification of IgE-binding proteins

## Abstract

New protein-rich flour blends from legumes are increasingly being processed for gluten-free bakery products, which may increase the risk of IgE-mediated sensitization through inhalation exposure. In the described case of a 29-year-old baker, work incapacity occurred due to respiratory complaints following exposure to various gluten-free baking ingredients. Occupation-related sensitization to “chestnut pea flour” (flour from lightly roasted and ground yellow peas) was identified, while the patient showed no IgE sensitization to five other ingredients. Significant allergic respiratory reactions occurred during bronchial challenge test with “chestnut pea” extract. Based on the test results and typical workplace-related symptoms, recognition of occupational disease was recommended. Further identification of potential causal allergens using IgE blot and allergen component-resolved diagnostics revealed IgE bindings to typical storage proteins, 2S albumin and vicilin, as well as to Bet v 1 homologous of “chestnut pea” and non-specific lipid transfer proteins. This case illustrates that the processing of protein-rich legume flours can pose new sensitization risks for workers, which should be considered in the future.

## Introduction 

As a trend towards sustainable nutrition and an alternative for gluten intolerance, protein-rich flours from legumes (*Leguminosae*) are increasingly being processed in the baking industry. According to the German Federal Statistical Office, the number of people purchasing gluten-free foods is steadily increasing. In 2023, ~ 3% of the German population, which equates to ~ 2.5 million people, were buying gluten-free products [1]. Legumes and nuts are also used in gluten-free bakeries to make the most of the nutty flavors and gluten-free properties of both flours. So called “Chestnut pea flour” is made from lightly roasted and ground yellow peas. It is used in baking to add protein, texture, and freshness to bread and other baked goods. This flour is particularly valued for its nutty flavor and ability to enhance the moisture retention of baked products. According to the EU Food Information Regulation No. 1169/2011, which is also applied by the Federal Institute for Risk Assessment, certain legumes such as peanuts and soybeans, and nuts such as almonds, hazelnuts, walnuts, cashew nuts, pecans, Brazil nuts, pistachios, and macadamia nuts must be declared due to their allergenic potential. Other protein-rich flours, such as pea flours, do not need to be explicitly declared. 

## Case report 

A 29-year-old atopic male baker (occasional smoker) presented for medical evaluation with respiratory symptoms during the production of gluten-free breads on a bakery scale. He had been working in the baking industry since 2011, but health problems only emerged in a large bakery when handling gluten-free products, particularly during dough preparation. Since April 2023, the patient had been unable to work due to respiratory and skin symptoms. Anamnestically, progressive, exclusively workplace-related shortness of breath had been reported since 2022, with mild complaints during the processing of psyllium husk and pronounced complaints when working with “chestnut pea flour”. Additionally, mild throat irritation occurred during rye milling, as well as itchy hand eczema during dough preparation activities. Initially, he was symptom-free in his home environment and on vacation, except for pollen-related seasonal rhino-conjunctivitis in summer. Over time, he developed pronounced perennial bronchial hyperreactivity outside of work as well. Both physical examination findings and general blood tests revealed no indicative findings. Baseline lung function testing was unremarkable, without manifest obstruction, and the methacholine test showed moderate bronchial hyperreactivity. Fractional exhaled nitric oxide (FeNO) was unremarkable at < 25 ppb. 

### Allergological diagnostics 

**Preparation and analysis of allergen test extracts from workplace samples**

**Figure 1.**
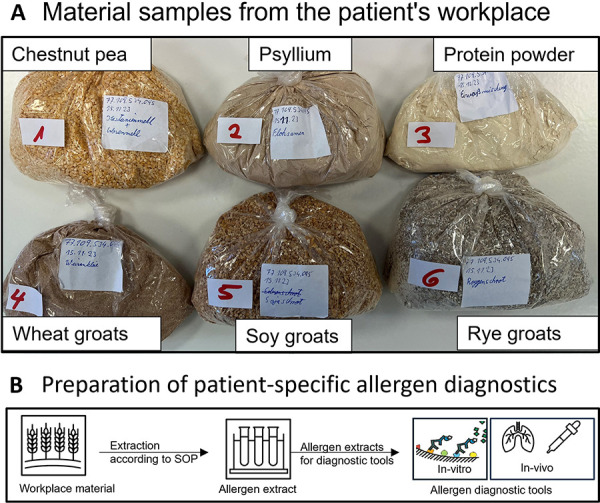
Six material samples from the patient’s workplace (A) and workflow (B) for the preparation of patient-specific allergen diagnostics.

Very few test substances are available for diagnostics of suspected occupational inhalation allergy. Since no standardized allergen test extracts are commercially available for the bakery products the patient handled at work, specific antigens were extracted from samples taken from the patient’s workplace (Figure 1) using neutral and ethanolic extraction methods established at the Institute for Prevention and Occupational medicine of the German social insurances (IPA) and described elsewhere [3]. Test solutions for in-vitro and in-vivo allergy diagnostics of the patient were prepared from the obtained allergen extracts of “chestnut pea flour”, psyllium, protein powder, wheat groats, soy groats, and rye groats. 

**Figure 2.**
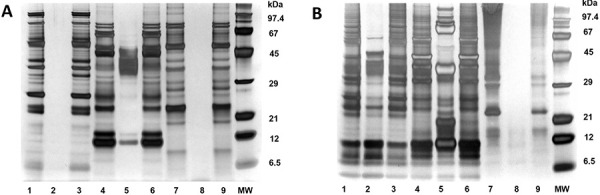
Silver staining of extracted proteins from six workplace samples of the employee in SDS-PAGE, applied protein amount per lane: 1.5 µg. Samples applied as aqueous and ethanolic extracts, as well as extracts for skin prick testing A 1 – 3: “chestnut pea”, A 4 – 6: protein powder, A 7 – 9: soy groats, B 1 – 3: wheat groats, B 4 – 6: rye groats, B 7 – 9: psyllium. MW = molecular weight protein standard.

The quality analysis of the allergen extracts from the workplace samples was performed quantitatively using the Bradford assay and qualitatively using SDS-silver-PAGE [3]. The extracted protein concentrations ranged from 0.26 mg/mL (psyllium) to 17.5 mg/mL (“chestnut pea flour”). Qualitatively, the protein patterns were analyzed in SDS-silver-PAGE with 1.5 µg of protein per lane. The allergen extracts from “chestnut pea”, soy groats, and psyllium showed only proteins in the neutral/water-soluble fraction (Figure 2A lanes 1+7, Figure 2B lane 7). Additional proteins were detected in the ethanolic fraction of the grain extracts and protein powder (Figure 2A lane 5, Figure 2B lanes 2+5). For ImmunoCAP (Thermofisher Scientific, Phadia AB, Uppsala, Schweden) coupling, these extracts were biotinylated according to standard procedures and then coupled to streptavidin ImmunoCAPs [9]. For extracts of psyllium, soy groats, and “chestnut pea”, only the neutral/aqueous fractions were used, while for wheat and rye meal and protein powder, a mixture of six parts neutral extract plus one part of ethanolic extract was used (Figure 2A lanes 1, 6, 7, Figure 2B lanes 3, 6, 7). The protein concentration was adjusted to 1 – 2 mg/mL. By coupling the biotinylated allergens to streptavidin ImmunoCAPs, specific allergen test tools for serological IgE determination were created. Similarly, allergen extracts were prepared in sterile packaging for skin prick testing (0.9% NaCl / 50% glycerine) and bronchial provocation testing (0.9% NaCl). 

**Results of in-vitro and in-vivo allergy testing**

To investigate the suspicion of an allergy-induced obstructive airway disease (including rhinopathy) in accordance with occupational disease number 4301 according to German legislation, a standard skin prick test using commercial allergen extracts form ALK (Hamburg, Germany), Leti (Ismaning, Germany), HAL Allergy (Düsseldorf, Germany), and Bencard (Munich, Germany) was initially performed, showing positive skin reactions to house dust mites (ALK), tree pollen (tree pollen mix 1: birch, alder, hazel, beech, oak, HAL Allergy), and grass pollen (*Phleum pratense*, ALK) (Table 1). This sensitization status was also confirmed serologically by ImmunoCAP system with a significantly elevated total IgE concentration of 778.57 kU/L and a positive inhalation allergen screen (sx1) (Table 1). Overall, multiple sensitizations to ubiquitous inhalation allergens were measured serologically and in commercial skin prick tests, but not to classical baker allergens such as wheat, rye, barley flour, or baking enzymes. A significantly elevated IgE concentration (2.73 kUA/L, CAP class 2) was measured against “chestnut pea” from the allergen extracts of the six workplace samples, prepared at IPA as described above, while the other five workplace samples showed no elevated IgE concentrations (Table 1). 

In the skin prick test with extracts from the workplace samples, the strongest reaction was also detected to “chestnut pea” extract (Table 1), with discrete reactions to protein powder and soy groats. To assess a workplace-related allergic obstructive airway disease in accordance with occupational disease number 4301, an indication for a bronchial provocation test with “chestnut pea” extract was present. 

Since the patient showed strong reactions to house dust mite both in the skin prick test and serologically (Table 1), all allergen extracts from the workplace samples were checked for possible contamination with mites. This was done using a quantitative polyclonal sandwich ELISA against mites [10], which showed that no mite antigens could be detected in three of the six workplace samples, namely “chestnut pea”, psyllium, and rye. Minimal mite antigen contamination was measurable in the other three workplace samples. Protein powder and soy groats contained ~ 20 ng mite antigen/mg substance, and wheat groats ~ 40 ng/mg, corresponding to 0.002% and 0.004% mite antigen in the respective workplace samples. These mite contaminations could possibly explain the discrete skin reactions to protein powder and soy groats. 

**Figure 3.**
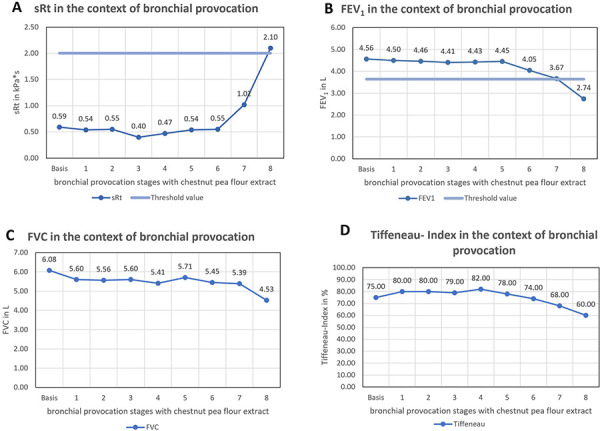
Progression of spirometric parameters after bronchial challenge testing with “chestnut pea extract” with increasing concentrations. A: Specific airway resistance; B: Forced expiratory volume in 1 second (FEV_1_), C: Forced vital capacity (FVC), D: Tiffeneau index (FEV_1_/FVC ratio), normal range is 70 – 80%.

The baseline examination on the first day showed moderate bronchial hyperreactivity after methacholine provocation without manifest obstruction. Exhaled FeNO was within the normal range at 16 ppb, and spirometry showed no pathological findings. The proportion of eosinophilic granulocytes in induced sputum was 23%. The bronchial provocation test was performed with “chestnut pea” extract in eight dilution stages, starting with 0.9% NaCl as the base, stage 1: 0.00018 mg/mL, increasing in 10-fold concentration steps up to stage 5: 1.8 mg/mL, stages 6 – 8 are cumulative doses of 1.8 mg/mL in each case (Figure 3). Spirometric and body plethysmographic criteria for a positive provocation test were met after the last stage, with specific airway resistance (sRt) increasing from 0.59 to 2.10 kPa×s (Figure 3A). The forced expiratory volume in one second (FEV_1_) decreased from 4.56 to 2.74 L (Figure 3B). Significant decreases were also observed in vital capacity (Figure 3C) and Tiffeneau index (Figure 3D). The examination 24 hours after provocation showed no increased bronchial hyperreactivity compared to the pre-measurement state, and the FeNO value remained almost unchanged. The percentage of eosinophilic granulocytes in induced sputum increased significantly from 23 to 36% after provocation. 

Based on these findings, recognition of occupational disease number 4301 (obstructive airway disease caused by allergenic substances, including rhinopathy) was recommended. The occupational disease-related reduction in earning capacity was assessed at 20% due to symptomatic bronchial hyperreactivity. As a preventive measure, the insured patient left his workplace and will change his career by requalification. 

**Identification of IgE-binding proteins**

**Figure 4.**
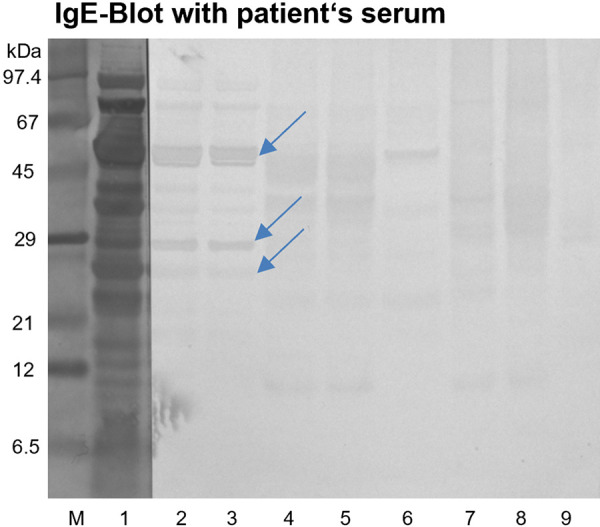
sIgE blot with serum from the exposed individual on baking ingredients that were positive in the skin prick test: lane M + 1: Coomassie-stained PVDF membrane, M: protein standard (10 µg), 1: “chestnut pea flour” (26 µg), lanes 2** – **9: IgE blot 2: “chestnut pea flour” (26 µg), 3: “chestnut pea flour” (13 µg), 4: neutral protein mix (27 µg), 5: neutral/ethanolic protein mix (27 µg), 6: neutral soy groats (17 µg), 7: neutral/ethanolic wheat groats (25 µg), 8: neutral/ethanolic rye groats (28 µg), 9: neutral psyllium (1.5 µg).

To identify relevant allergens in the workplace samples of the employee, a Western blot was performed with all extracts, and IgE bindings to specific protein bands were detected using a standard procedure [4] with an anti-human IgE antibody (Figure 4). Overall, the IgE blot showed weak bands in all extracts, with three protein bands marked on “chestnut pea” extract at molecular weights of 25, 28, and 50 kDa. These three bands were excised from a corresponding Coomassie-stained SDS-PAGE and sent for mass spectrometric analysis (Proteome Factory AG, Berlin, Germany). The obtained peptide sequences were compared against the SwissProt and the plant subset of RefSeq databases. For two of the three protein bands, a protein was identified based on the emPAI values (60.04 and 15.31) and a match of at least 80% of the peptide sequences, indicating a significantly higher presence than other proteins. The 25-kDa protein from “chestnut pea” was identified as Albumin-2 OS=Pisum sativum OX = 3888 PE = 2 SV = 1 (SwissProt), 26.393 kDa, pI 5.16. The 50-kDa protein band was identified as Vicilin OS = Pisum sativum OX = 3888 PE = 2 SV = 2 (SwissProt) with a mono-isotopic mass of 52.257 kDa and a calculated pI of 5.4. The third protein band from “chestnut pea” could not be definitively identified. The identified pea proteins, 2S albumins and vicilins (7S globulins), are typical storage proteins in legumes and nuts. Numerous cross-reactive food allergens from these two protein families have already been identified, and their allergological significance has been described in the position paper of the German Society of Allergology and Clinical Immunology (DGAKI) Food Working Group [7]. Currently, five allergens are known for peas (Table 2). The 50-kDa pea vicilin identified could represent an isoform or fragment of the major allergens Pis s 1 or Pis s 2 [8]. 2S albumins from peas have not yet been described as allergens but are considered potentially non-relevant food allergens, unlike other 2S albumins from peanuts (Ara h 2) or cashews (Ana o 3) [5, 7]. 

**Analysis of individual allergens and potential cross-reactive allergens**

Using a serological analysis of individual allergen components for the “chestnut pea”-sensitized patient in the ALEX Allergy Explorer test (MacroAssay Diagnostics, Vienna, Austria), the results of allergen identification from the IgE blot should be verified and potential cross-allergens identified. Surprisingly, the ALEX test did not measure elevated sIgE concentrations against storage proteins (2S albumins, 7/8S globulins, 11S globulins) from legumes, nor against the total extract of chickpea, which was also available on the array. Instead, a total of 25 individual allergens with sIgE concentrations > 0.35 kU/L (Table 3) were measured, with particularly high sIgE concentrations against the major allergen from birch pollen (Bet v 1) and Bet v 1 homologous. Elevated sIgE concentrations against food allergens were exclusively measured against four Bet v 1 homologs in celery, peanut, soy, and apple, as well as against three non-specific lipid transfer proteins in peanut, strawberry, and peach (Table 3). 

Other values, such as the total IgE content in the ALEX Allergy Explorer, were approximately twice as high at 1,465.89 kU/L compared to the ImmunoCAP system. In addition to mite allergens Der f 2, Der p 2, and Der p 23, the serological IgE concentrations against Bet v 1 homologs were the highest in the patient. Accordingly, the Bet v 1 homologs from pea, Pis s 6, could act as the causal allergen during the processing of pea flour. It has been described that Bet v 1 homologous allergens from legumes can cause severe allergic reactions in subjects suffering from birch pollen allergy, for example, when consuming soy drinks [11]. Similarly, atopic individuals with tree pollen allergy and Bet v 1 sensitization are advised to avoid consuming large amounts of soy products and massive exposure to unprocessed soybeans, especially in the workplace [2]. In the patient described here, asthmatic symptoms at the workplace could possibly be induced by exposure to Bet v 1 homologs in “chestnut pea flour”. Whether the respiratory occupational symptoms were causally induced by storage proteins 2S albumins or vicilins, or by Bet v 1 homologs from pea could not be conclusively determined. Further investigations should clarify the impact of inhalation exposure to typical food allergens, such as storage proteins from legumes, as the primary cause of IgE-mediated sensitization. 

## Summary and conclusion 

New protein-rich flour blends from legumes are increasingly being processed in bakeries. Consequently, the risk of IgE-mediated sensitization through inhalation exposure to legume allergens in the workplace is expected to rise and should be considered in the future. In the case described here, occupational sensitization to “chestnut pea flour” was initially identified through the production of standardized allergen extracts in a stepwise approach. This allowed five out of six workplace materials to be excluded from further diagnostics. The workplace-related inhalation test, as the gold standard for assessing occupational obstructive airway disease [6], was conducted in this case through cumulative increases in the extract-based allergen dose during bronchial provocation testing. This approach helped reduce the risk of severe bronchial and systemic allergic reactions. 

Based on the investigation results and typical workplace-related symptoms, recognition of occupational disease number 4301 was recommended for the patient. This case also illustrates that the introduction and processing of protein-rich flours from legumes, specifically “chestnut pea”, can lead to new sensitization risks for exposed workers. 

## Authors’ contributions 

Conception and design of the study: Who was responsible for the idea and design of the study? SK, CE, MR. 

Data collection: Who collected the data or conducted the experiments? SM, IM, CE, SK. 

Data analysis and interpretation: Who analyzed the data and interpreted the results? SK, CE, IM, IS, MR. 

Manuscript drafting: Who wrote the manuscript and who reviewed it? SK, CE, IS, IM, MA, MR. 

## Funding 

No special funding required. 

## Conflict of interest 

IM and MA are employees of MacroArray Diagnostics. All other authors declare that they have no conflict of interest with regard to this work. 

**Table 1.** IgE sensitization tests in vitro (ImmunoCAP) and in-vivo (skin prick test).

**Allergen**	IgE concentration*	Skin prick test** **(wheal/erythema)**
“Chestnut pea”	**2.73 kU_A_/L; CAP-class 2**	**9 / 43 mm**
Protein powder	0.20 kU_A_/L; CAP-class 0	**4 / 30 mm**
Soy groats	0.18 kU_A_/L; CAP-class 0	**4 / 22 mm**
Wheat groats	0.12 kU_A_/L; CAP-class 0	2 / 3 mm
Rye groats	0.14 kU_A_/L; CAP-class 0	2 / 4 mm
Psyllium	0.02 kU_A_/L; CAP-class 0	2 / 2 mm
Inhalation screen (sx1)	**44.14 kU** **_A_** **/L; positive**	**10 / 20 mm**
Gras pollen mix (gx 1)	**2.53 kU** **_A_** **/L; positive**	**4 / 13 mm** (tree pollen)
Domestic mite (d1)	**20.47 kU** **_A_** **/L; CAP-class 4**	**20 / 45 mm** (*Phleum pratense*)
Flour mite (d70)	**2.53 kU** **_A_** **/L; CAP-class 2**	nd
Total IgE (tIgE)	**778.57 kU/L; increased**	**Histamine 7 /30 mm

*Serological sIgE concentration < 0.35 kUA/L: wheat flour (f4), rye flour (f5), barley flour (f6), soy (f14), alpha-amylase nAsp o 21 (k87), gluco-amylase *A. niger* (U296), xylanase *A. niger* (bg226), cellulase *A. niger* (bg236).

**Table 2.** llergens from pea (*Pisum sativum*).

**Allergen**	**Allergen family**	**Molecular weight**
Pis s 1	7S-viciline	44 kDa
Pis s 2	7S-viciline/convicilin	63 kDa
Pis s 3	nsLTP	9.5 kDa
Pis s 5*	profilin	14 kDa
Pis s 6*	Bet v 1- homologous	17 kDa

*Not classified according to WHO/IUIS but www.allergome.org.

**Table 3.** Specific IgE sensitization to single allergens by ALEX Allergy Explorer array.

**Allergen**	**Organism**	**Species**	**IgE-reactivity Alex-Chip [kUA/L]**	**Allergen family **
**Aln g 1**	Alder	*Alnus glutinosa*	**44.31**	**Bet v 1 homolog, PR-10**
**Api g 1**	Celery	*Apium graveolens*	**1.74**	Bet v 1 homolog, PR-10
**Ara h 8**	Peanut	*Arachis hypogaea*	**7.67**	Bet v 1 homolog, PR-10
**Bet v 1**	Silver birch	*Betula verrucosa*	**46.54**	Bet v 1 family, PR-10
**Cor a 1.0401**	Hazelnut	*Corylus avellana*	**15.35**	Bet v 1 homolog, PR-10
**Gly m 4**	Soy	*Glycine max*	**4.32**	Bet v 1 homolog, PR-10
**Mal d 1**	Apple	*Malus domestica*	**8.08**	Bet v 1 homolog, PR-10
**Que a 1**	Oak	*Quercus alba*	**29.87**	Bet v 1 homolog, PR-10
				
**Ara h 9**	Peanut	*Arachis hypogaea*	**1.80**	**nonspecific LTP type 1**
**Fra a 3**	Strawberry	*Fragaria ananassa*	**0.36**	nonspecific LTP type 1
**Pla a 3**	London plane tree	*Platanus acerifolia*	**0.76**	nonspecific LTP type 1
**Pru p 3**	Peach	*Prunus persica*	**1.28**	nonspecific LTP type 1
				
**Bla g 9**	German Cockroach	*Blatella germanica*	**36.45**	**Arginine kinase**
**Der p 20**	European house dust mite	*Dermatophagoides pteronyssinus*	**12.44**	Arginine kinase
**Pen m 2**	Black Tiger Shrimp	*Penaeus monodon*	**14.62**	Arginine kinase
				
**Phl p 1**	Timothy grass	*Phleum pratense*	**3.74**	**Beta-expansin**
**Zea m 1**	Maize	*Zea mays*	**1.40**	Beta-expansin
				
**Der f 1**	American house dust mite	*Dermatophagoides farinae*	**0.40**	**Cysteine protease**
**Der p 1**	European house dust mite	*Dermatophagoides pteronyssinus*	**6.36**	Cysteine protease
				
**Amb a 4**	Ragweed	*Ambrosia artemisiifolia*	**0.74**	**Defensin-like protein**
**Art v 1**	Mugwort	*Artemisia vulgaris*	**3.48**	Defensin-like protein
				
**Der f 2**	American house dust mite	*Dermatophagoides farinae*	**34.69**	**NPC2 family**
**Der p 2**	European house dust mite	*Dermatophagoides pteronyssinus*	**39.82**	NPC2 family
				
**Der p 23**	European house dust mite	*Dermatophagoides pteronyssinus*	**30.83**	**Peritrophin-like protein domain **
				
**Api m 1**	Honey bee	*Apis mellifera*	**0.71**	Phospholipase A2
